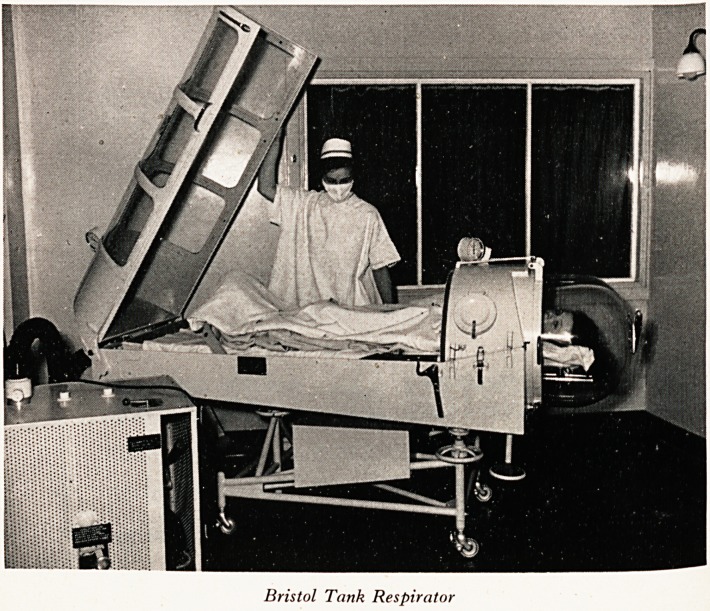# Respirator Research at Ham Green Hospital

**Published:** 1954-07

**Authors:** 


PLATE XIII

*Clevedon Positive-Pressure Respirator*

PLATE XIV

*Bristol Tank Respirator*

### Editorial

### RESPIRATOR RESEARCH AT HAM GREEN HOSPITAL

Breathing difficulties in poliomyelitis are most commonly due either to weakness of the respiratory muscles or to obstruction of the upper respiratory passages by secretions. The first group will usually respond to treatment in a tank-type of respirator and the second to postural drainage combined with suction. There remains a group of cases with mixed spinal and bulbar involvement which had a very high mortality until two years ago when Lassen[1] introduced a new method of treatment during the severe epidemic of poliomyelitis in Copenhagen. This involves a tracheotomy with insertion of a cuffed tube which blocks off the pharynx and its secretions and through which the lungs are regularly inflated by positive pressure. Lassen used relays of medical students to supply the pressure.

Working at Ham Green, Macrae and McKendrick have developed an automatic machine, the *Clevedon Positive-Pressure Respirator* (Plate XIII), to take the place of the medical students and have reported in the *Lancet* their experience with their first case.[2] The patient, a man of twenty-one, would undoubtedly have died without this new method. Progress has so far been very satisfactory, power of swallowing returning after six days and some spontaneous respiration beginning soon after. Forty-seven days after the start of positive-pressure respiration the tracheotomy tube was finally removed and the wound allowed to heal. The patient has now been home for more than a month and is able to walk and look after himself.

It is believed that this is the first case of poliomyelitis to be treated by this method in this country. Although the difficulties of management have not been so great as was feared close supervision is vital and at Ham Green it has been considered essential to have a doctor on the ward twenty-four hours a day. The method is therefore one which can only be used in well-equipped hospitals with adequate medical staff.

Many difficulties have had to be overcome to produce a satisfactory respirator within a few months and the workers at Ham Green are to be congratulated.

Last year it was also apparent that the manufacture and design of tank respirators in this country had not moved appreciably since Lord Nuffield presented the *Both* respirator to the Nation in 1938. It seemed, therefore, appropriate to try to utilize the wealth of mechanical skill available in the Bristol area to design and build a prototype tank respirator on entirely new lines.

The subject was studied at Ham Green, and the co-operation of the Bristol Aeroplane Company was sought and readily obtained. In August, 1953, the South Western Regional Hospital Board authorized the necessary expenditure, and the apparatus was completed in December.

The *Bristol Tank Respirator* (Plate XIV) is now installed at Ham Green Hospital and has already been used in the care of several patients.

The primary object of the design is ease of nursing combined with comfort for the patient. Construction is largely in light alloy and perspex, and a pleasing appearance has been obtained quite unlike any previous type of tank respirator. The machine provides for positive-pressure breathing through the mouth as well as conventional negative-pressure cabin respiration, and longitudinal and lateral tilt can be simply and accurately adjusted. The speed of breathing and volume of tidal air can be varied within wide limits.

The development of this machine owes much to the interest and enthusiasm of members of the staff of the Bristol Aeroplane Company. It has been very successful in use and one has been ordered for use in the Bristol United Hospital.[3]

## Figures and Tables

**Figure f1:**
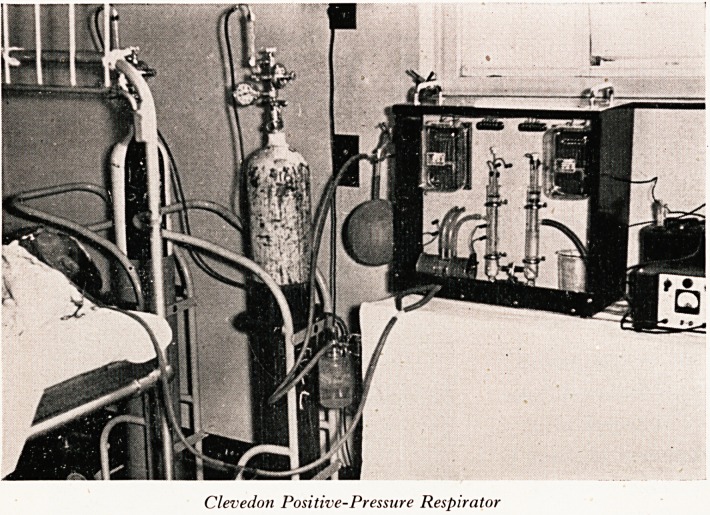


**Figure f2:**